# Optimization of Ultrasound-Assisted Extraction of Glucosinolates from Upcycled Cauliflower Using Response Surface Methodology

**DOI:** 10.3390/molecules30112326

**Published:** 2025-05-26

**Authors:** Hana Derbew Gedif, Tess Astatkie, Joanna Tkaczewska, H. P. Vasantha Rupasinghe

**Affiliations:** 1 Department of Plant, Food, and Environmental Sciences, Faculty of Agriculture, Dalhousie University, Truro, NS B2N 5E3, Canada; hn271655@dal.ca; 2 Department of Animal Product Technology, Faculty of Food Technology, University of Agriculture, Balicka 122, 30-149 Kraków, Poland; 3 Faculty of Agriculture, Dalhousie University, Truro, NS B2N 5E3, Canada

**Keywords:** *Brassica oleracea*, sulforaphane, green extraction, food waste, antioxidants, UPLC-ESI-MS

## Abstract

This study aimed to optimize the ultrasound-assisted extraction (UAE) process using food-grade ethanol to recover glucosinolates from upcycled cauliflower through response surface methodology. The optimized extraction process was compared with traditional extraction using maceration with solvents such as methanol and acetone. The optimum UAE conditions identified for extracting glucosinolates from upcycled cauliflower were: 42% ethanol as solvent at 43 °C for 30 min. The total glucosinolate content recovered was 7400 μg sinigrin equivalence (SE)/g dry weight (DW) of biomass. The ultra-pressure liquid chromatography–electrospray ionization-mass spectrometry (UPLC-ESI-MS) analysis confirmed that the optimized UAE yielded the highest levels of glucoraphanin (1.31 ± 0.12 μg/g DW of biomass) and sulforaphane (28.2 ± 3.34 μg/g DW of biomass). The extracts possess greater antioxidant activity as determined by ferric reducing antioxidant power and DPPH radical scavenging activity. The optimized UAE process significantly enhanced the extraction of valuable phytochemical molecules from the upcycled cauliflower. Further studies should focus on evaluating their therapeutic and preventive potential for applications in nutrition and health.

## 1. Introduction

Glucosinolates (GLS) are secondary metabolites ubiquitous in the Brassicaceae family. GLS consist of variable side chains produced from amino acids, a D-thioglucose unit, and a sulfonated aldoxime domain [1,2]. Over 120 distinct GLS have been identified in various plants, mainly cruciferous vegetables including cauliflower (*Brassica oleracea* cultivar Botrytis group), broccoli (*Brassica oleracea* var. italica), mustard (*Sinapis alba*), cabbage (*Brassica oleracea* cultivar Capitata group), and radish (*Raphanus sativus*) [3]. The composition and concentration of GLS differ depending on the species, growing environment, variety, and plant age [1,3,4]. Recent studies have demonstrated that GLS exhibit a range of health benefits, including antioxidant, anti-inflammatory, anti-cancer, and cardio-protective activities [5,6,7]. The main types of GLS found in cauliflower are glucoraphanin, glucobrassicin, glucoiberin, neoglucobrassicin, and glucoerucin [8].

Cauliflower, one of several vegetables in the Brassica family, is widely consumed worldwide. Cauliflower provides a substantial contribution to the human diet due to its diverse bioactive phytochemicals with potential health benefits [9]. The major phytochemicals of cauliflower are glucosinolates, polyphenols, and carotenoids [10]. It has been shown that a regular consumption of cruciferous vegetables lowers the risk of several diseases, including some cancers (e.g., colon, prostate, and breast), cardiovascular diseases, and obesity [11,12].

Ultrasonication-assisted extraction (UAE) has emerged as an effective and environmentally friendly technique for extracting phytochemicals from diverse plant materials, providing increased efficiency while maintaining chemical integrity under normal processing conditions [13,14]. UAE can be used to break down cell walls, thereby enabling an enhanced interaction between extraction solvents and phytochemicals. Moreover, the use of UAE has been shown to enhance the efficiency of extraction at low temperatures, which is crucial for preventing the thermal degradation of phytochemicals [15]. However, the major disadvantage is the unavailability of commercial UAE systems to handle a large volume of biomass compared to traditional extraction methods such as maceration. Organic solvents with varying polarities, including ethanol, hexane, methanol, acetonitrile, and acetone, are commonly used to extract phytochemicals from various plant species [11]. However, food-grade ethanol is chosen over other solvents for extracting bioactive phytochemicals due to its low toxicity and ability to extract a wide range of phytochemicals [15]. The present study aims to determine the optimum UAE conditions for maximizing glucosinolate recovery from upcycled cauliflower. Therefore, the glucosinolate extraction method for cauliflower was optimized by central composite design (CCD) combined with response surface methodology (RSM) for three initial extraction parameters: % ethanol, extraction temperature, and extraction time. The total antioxidant activity of extracts obtained by conventional solvents versus the optimized UAE–ethanol was compared.

## 2. Results and Discussion

### 2.1. Response Surface Analysis and Optimization

UAE is an emerging extraction method that offers several benefits, including faster extraction of phytochemicals from natural sources, simpler operation, reduced solvent usage, and greater reproducibility with lower temperature and energy requirements [16]. In the present study, CCD, a response surface design with 20 runs, was used to optimize the glucosinolate content from cauliflower extracts, and the results are presented in Table 1. Three extraction factors were considered: % ethanol, extraction temperature, and time; and the obtained data were used to determine the ideal setting for upcycled cauliflower powder extract, focusing on achieving high total glucosinolate content (TGC), total phenolic content (TPC), and total carotenoid content (TCC). The analysis of variance (ANOVA) results that indicate significant variation (*p* ≤ 0.05) in the observed values of each variable at various combinations of ethanol concentration, extraction temperature, and time are shown in Table 2. The TPC of cauliflower under UAE conditions was significantly affected by the main effect (% ethanol) and quadratic effects (% ethanol and time). Similarly, all the quadratic terms showed a significant positive effect on TCC. As shown in Table 2, the adjusted R^2^ values for TPC, TCC, and TGC (67.93%, 69.34%, and 57.52%, respectively) indicate that the model captures the variability in TPC, TCC, and TGC moderately. However, since the lack of fit *p*-values were all higher than 5% (the level of significance used), the data fit the model very well.

The results indicated that extraction in lower ethanol contents (≤50%), extraction temperature (≤50 °C), and extraction time less than 40 min results in comparatively higher glucosinolate content. In contrast, increases above these levels resulted in a significant decrease in yield. Mild heating can weaken the plant tissue, reduce the cell walls’ strength, and enhance phytochemicals’ solubility, allowing more compounds to dissolve into the solvent. However, phytochemicals may be degraded by prolonged exposure to ultrasonic waves at higher temperatures [16]. Moreover, increasing the solvent concentration at a specific level causes polarity differences and affects its dissipation factor, encouraging the efflux of phytochemicals from the cell matrix [17].

The fitted models that allow the prediction of the three response variables at a given value of the three factors are:

TGC (µg SE/g DW) = 15.6 + 0.360 A + 0.409 C + 0.82 B − 0.00417 A × A − 0.00652 C × C − 0.0136 B × B − 0.00622 A × C + 0.00422 A × B + 0.0057 C × B

TCC (µg/g) β−carotene = −22.22 + 0.1329 A + 0.440 C + 0.526 B − 0.001237 A × A − 0.00414 C × C− 0.00465 B × B − 0.00041 A × C + 0.00023 A × B − 0.00147 C × B

TPC (GAE (µg)/g DW) = −0.1458 + 0.002115A + 0.00376C + 0.00479 B − 0.000013 A × A − 0.000029 C × C − 0.000034 B × B− 0.000006 A × C − 0.000006 A × B − 0.000025 C × B

A, B, and C represent the ethanol content, extraction temperature, and extraction time, respectively.

**Figure 1:**
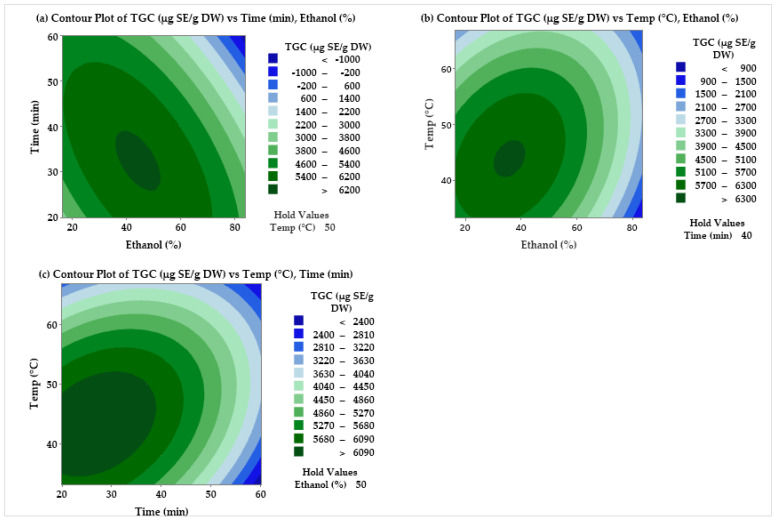
Contour plots of total glucosinolate content (µg/g DW) vs. (**a**) time (min) and ethanol (%) with temperature held at 50 °C, (**b**) temperature (°C) and ethanol (%) with time held at 40 min, and (**c**) temperature (°C) and time (min) with ethanol held at 50%.

Three-dimensional (3D) response surface plots were used to show the main and interaction effects of the three factors and confirm the optimal conditions on the TGC, TPC, and TCC of the extract (Figure 1). The optimum value of ethanol content, extraction temperature, and time that give the maximum discovery of the extract phytochemical properties were found to be: TGC—42% at 43 °C and 30 min; TPC—59% at 50.8 °C and 36 min; and TCC—51.69% at 51.18 °C and 41.42 min. The experimentally obtained values for the response variables are as follows: 7474 µg SE/g DW for total glucosinolate content, 195.3 µg GAE/g DW for total polyphenols, and 4.16 µg CE/g DW for total carotenoid content. The corresponding predicted values at the optimal setting are TGC: 9448 µg SE/g DW; TPC: 156.4 GAE µg/g DW; and TCC: 3.05 µg CE/g DW. The extraction experiment was carried out under the optimal TGC conditions to confirm the accuracy of the model equation. Therefore, the experimentally determined values are in good agreement with the predicted total glucosinolate values by the RSM, and hence, the model fitness is highly relevant for the specified range of the control variable. However, the adjusted R^2^ value for TGC of 57.52 of the model indicates limited explanatory power of the model for TGC. Therefore, some uncontrolled factors, such as ultrasound power and solid–liquid ratio, may have affected the results, and in future studies, these additional factors need to be investigated. Moreover, the synergistic effect of ultrasound cavitation and solvent polarity on the efficiency of glucosinolate extraction needs to be investigated. For example, there could be a correlation between ethanol concentration and the degree of cell wall damage, thus, the recovery of TGC.

### 2.2. CCD for Optimization of UAE Conditions

**Figure 2:**
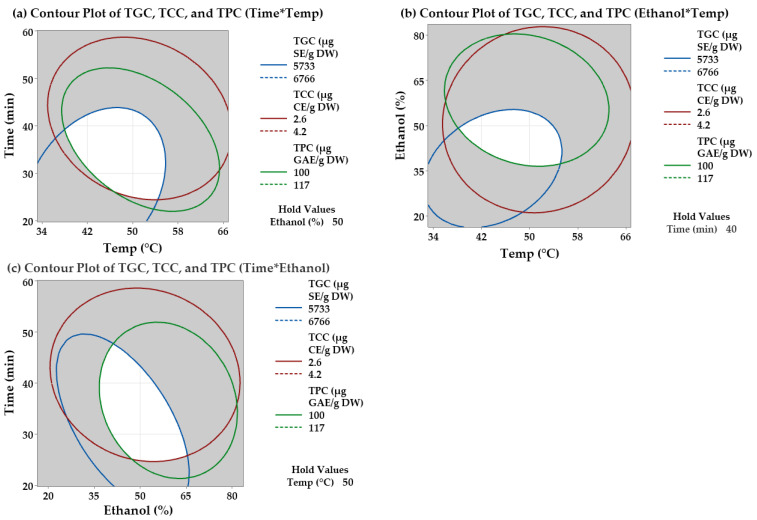
Overlaid contour plot that shows the sweet spot of cauliflower extract at different ethanol concentrations, extraction time, and temperature: TGC (µg/g), TPC (GAE µg/g DW) and TCC (µg/g DW): (**a**) Time and Temperature with Ethanol held at 50%, (**b**) Ethanol and Temperature with Time held at 40 min, (**c**) Time and Ethanol with Temperature held at 50 °C.

Contour plots were created to investigate the effect of selected extraction factors on TGC, TPC, and TCC of cauliflower extract (Figure 1 and Figure 2). These plots have shown how TGC changed as ethanol concentration and extraction time changed while the extraction time was kept constant at 40 min (Figure 1a), as extraction time and temperature changed while the ethanol content was kept constant at 50% (Figure 1b), and as ethanol concentration and time changed while keeping the temperature constant at 50 °C (Figure 1c). Higher quantities of TGC were extracted from cauliflower extract (>62 mg SE/g DW) when the ethanol content was around 50%. A similar behavior was observed by Bojorquez-Rodríguez et al. [11] during the extraction of glucosinolates from broccoli sprouts. These authors indicated that the optimal condition was 50% ethanol/water (*v*/*v*), 40 °C, and 1:35 (*w*/*v*), and they obtained the highest yield of around 85% of total glucosinolates. Interestingly, TGC yield significantly dropped as ethanol content increased above 60% (*v*/*v*), and higher ethanol concentration had a significant impact on the total glucosinolates content. Extraction temperature and time also affect the total glucosinolates content, and higher TGC yield has been obtained when lower extraction temperature and time were used.

The overlaid contour plot shows the optimum settings of the three factors to maximize the phytochemical content (Figure 2). From these figures, high values of TGC range from 5733 to 6766 µg SE/g DW, high values of TPC range from 99.6 to 117 (g GAE/g DW, and high values of TTC range from 2.59 to 4.22 µg CE/g DW. Then, the overlaid contour plots (Figure 2) illustrate the sweet spot (white area) that represents the optimum conditions of all three response variables to maximize TGC, TPC, and TTC within these ranges in cauliflower. The optimal setting of % ethanol, extraction temperature, and time for cauliflower extract found through response optimizer and the overlaid contour plots were 50, 50, and 40, respectively.

### 2.3. Comparison of the UAE Extraction Method with Traditional Extraction Methods

#### 2.3.1. Phytochemical Profiles

**Figure 3:**
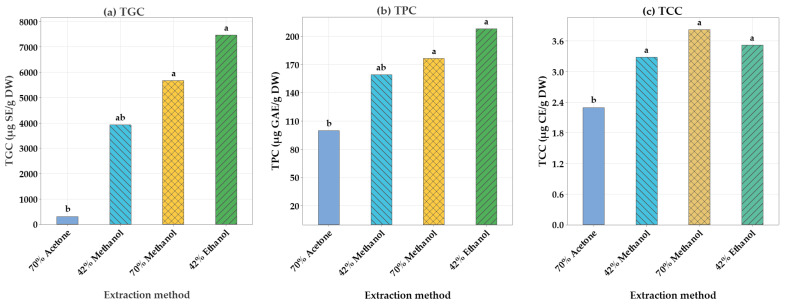
The total glucosinolate content (**a**), total phenolic content (**b**), and total carotenoid content (**c**), The total glucosinolate content is expressed in µg SE/g DW of the sample. Total phenolic content (µg GAE/g DW), and total carotenoid content (µg CE/g DW) were used for comparison purposes. Mean values with the same letter are not significantly different at the 5% level.

Comparison was carried out between UAE and traditional methods to evaluate the effectiveness of the optimized UAE methodology on the extraction of TGC, TPC, and TCC from cauliflower. Cauliflower extracts were prepared applying the optimal UAE conditions of the present study: 42% ethanol; Temp at 43 °C, 30 min, and 42% methanol; Temp at 43 °C, 30 min for UAE, and 70%. Conventional extraction method under similar conditions: Acetone; Temp at 70 °C, 20 min, and 70% methanol; Temp at 70 °C, 20 min. The comparative analysis of TGC, TPC, and TCC of the cauliflower extracts using ultrasonic extraction and traditional methods is shown in Figure 3. Compared to conventional extraction methods, the optimized UAE conditions (42% ethanol; Temp at 43 °C, 30 min) enabled the obtaining of cauliflower extract with higher TGC (7474 ± 103 µg/g DW) and TPC (208 ± 3.6 µg GAE/g DW). This could be because ultrasound power enhances mass transfer and extraction efficiency by decreasing external resistance during UAE. This is achieved by reducing the thickness of the boundary layer and creating a mixing process at the solid–liquid interface [18]. Moreover, polyphenols are sensitive components, and the thermal destruction of isolated compounds takes place at high temperatures [19], therefore, the lowest TPC was recorded for the traditional extraction method. A study by Okumus [20] showed that the flower of *Crambe tataria* had the highest content of sinigrin (690 mg/kg) when the ultrasonic extraction method was used compared to the traditional extraction method. The results were similar to those obtained by Yuan et al. [21], who used UAE as the extraction method and found that ethanol showed the highest total phenolic content (246 ± 6.41 mg GAE/g DW) of Propolis extract. Similarly, Vieira et al. [22] reported that UAE significantly increased the extracted total phenolic content (5.38 ± 0.28 mg GAE/g DW) of chayote leaf extracts compared to the maceration method (4.24 ± 0.41 mg GAE/g DW). Moreira-Rodríguez et al. [23] demonstrated the differences between methanol and ethanol (70:30 *v*/*v*) on broccoli sprouts extracts, finding that ethanol extracts contained higher levels of specific glucosinolates (33.3 ± 0.84 mmol/kg), such as glucoraphanin, while methanol extracts had slightly higher levels of phenolic compounds. The traditional methanol extraction method: (70% methanol; Temp at 70 °C, 20), obtained a higher TCC value of 3.82 ± 0.17 mg/g DW, while the traditional acetone extraction method (70% acetone; Temp at 70 °C, 20 min) obtained a significantly lower TCC value of 2.29 ± 0.64 mg CE/g DW. To further assess the advantages of UAE over traditional extraction methods such as maceration, additional comparisons need to be performed using the same solvent, such as 42% ethanol.

#### 2.3.2. Antioxidant Capacity

**Figure 4:**
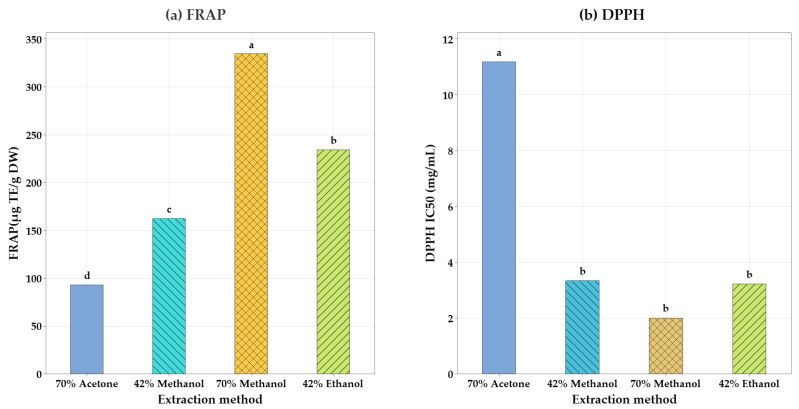
The total antioxidant capacity (FRAP) (**a**) and DDPH (**b**). The FRAP activity is expressed in μg TE/g; DW and DDPH (IC_50_ mg/mL) were used for comparison purposes. Mean values with the same letter are not significantly different at the 5% level.

Cauliflower wastes are rich in flavonoids, particularly kaempferol and quercetin, which have high antioxidant properties: flavonoids scavenge free radicals, thereby lowering oxidative stress and decreasing the risk of chronic diseases, including cancer and heart disease [24]. Figure 4 shows the FRAP and DDPH radical scavenging assays performed to compare the total antioxidant capacity using UAE and the traditional methods. A higher level of antioxidant activity is shown by a lower IC_50_ level (the amount of antioxidant required to reduce DPPH radical by 50%) [25]. The result indicated that the overall antioxidant capacity was significantly affected by traditional extraction methods as compared to the corresponding UAE methods. The 70% methanol solvent obtained the extracts with the maximum antioxidant activity according to the FRAP and DPPH measurements (Figure 4). The extracts obtained from 70% methanol, Temp at 70 °C, and 20 min had greater FRAP values (335 ± 22.2 μg TE/g DW) and a lower IC_50_ value, 2.01 ± 0.12 mg/mL (higher antioxidant activity), whereas the extract obtained with 70% acetone solvent showed a significantly higher IC_50_ value (11.2 ± 1.19 mg/mL) compared to other extracts produced by UAE. Antioxidant activity typically increases as phenolic concentration and the number of hydroxyl groups increase. The phenolic hydroxyl groups, being the primary reduction site, can react with the free radicals formed during the oxidation process as hydrogen donors [26]. UAE depends on cavitation, which enhances extraction, but it can also produce free radicals and cause degradation of sensitive antioxidant compounds. In addition, the free radicals produced during UAE may interact with antioxidant compounds, decreasing their total scavenging capacity [27].

#### 2.3.3. Glucosinolate Profile of Cauliflower Extracts Using UPLC-ESI-MS Analysis

The glucosinolate profiles by ultra-pressure liquid chromatography–electrospray ionization–mass spectrometry (UPLC-ESI-MS) for comparing the optimal extraction conditions with different extraction methods of upcycled cauliflower extracts are presented in Table 3. The results show the various glucosinolates present in upcycled cauliflower extracts, including glucoraphanin (μg/g) and sulforaphane (μg/g) compounds. The study found a significant difference (*p* < 0.05) for glucoraphanin (μg/g) and sulforaphane (μg/g) using the ethanol optimum extraction method compared to other extraction methods. Higher concentrations of glucoraphanin (μg/g) and sulforaphane (μg/g) were obtained in upcycled cauliflower by applying UAE extraction with 42% ethanol, resulting in (1.31 ± 0.12 μg/g DW of biomass) and (28.6 ± 3.34 μg/g DW of biomass), respectively. The findings agree with a prior study by Martínez-Zamora et al. [28], who performed the extraction of bioactive compounds from broccoli leaf and floret byproducts through UAE. The sulforaphane content obtained from UAE broccoli florets ranged from 2.5- to 4.5-fold higher compared to the leaf extracts obtained from the same plants. The acoustic cavitation effect in UAE accelerated the mass transfer and solubilization of the targeted compounds, increasing the glucoraphanin and sulforaphane of the cauliflower extract [29]. UAE appears as a green and effective way to extract glucosinolates from repurposed cauliflower. Its ability to maximize compound recovery while reducing solvent use makes it a promising approach for sustainable food waste valorization and bioactive compound production. Therefore, given the growing need for sustainable and environmentally responsible processes, it is crucial to consider green extraction techniques, which offer the potential to produce products with enhanced quality, improved functionality, lower energy consumption, and decreased waste generation [30].

## 3. Materials and Methods

### 3.1. Chemicals and Plant Material

Upcycled cauliflower powder was provided by Outcast Foods Limited, Dartmouth, NS, Canada. All the chemicals and reagents used in this research were purchased from Sigma-Aldrich (Oakville, ON, Canada).

### 3.2. Experimental Design

The extraction was performed through the Response Surface Methodology (RSM) using the Central Composite Design (CCD) to determine the optimal condition required to recover glucosinolates of cauliflower. The experimental factors were ethanol content (16.3%, 30%, 50%, 70%, and 83.5%), extraction temperature (33, 40, 50, 60, and 67 °C), and time (20, 28, 40, 52, and 60 min). The design generated 20 experimental runs, and RSM was used to analyze the effect of each process variable on total glucosinolates content (TTC), total polyphenol content (TPC), and total carotenoid content (TCC) (Table 1).

### 3.3. Ultrasound-Assisted Extraction (UAE)

All the UAE experiments were conducted using an ultrasonication bath [31]. UAE was performed by mixing upcycled and dried cauliflower powder (0.5 g) with the selected solvent (10 mL) in glass tubes and mixing properly for 1 min using a homogenizer (Sorvall ST 18 centrifuge, Thermo Scientific Inc., Ottawa, ON, Canada). These samples were subjected to UAE at different temperatures (33.1–66.8 °C) for various time ranges (28–52 min) [32]. Then, the extracts were centrifuged at 1000× *g* for 10 min, filtered, and stored at −20 °C until further analysis.

### 3.4. Traditional Extraction

The traditional extraction procedure was carried out according to a previously established method [33] with some modification. Briefly, each cauliflower powder sample (0.5 g) was mixed with 10 mL of methanol (70%) or acetone (70%) and mixed properly for 1 min using a vortex machine. These samples were kept in a shaking water bath at 70 °C for 30 min. Then, the extracts were centrifuged at 1000× *g* for 10 min, filtered, and stored at −20 °C until further analysis.

### 3.5. Total Glucosinolate Content (TGC)

The TGC of the cauliflower extracts was determined using a previously described method [24] with minor modifications. Briefly, cauliflower extract (2 mL) was mixed with 2 mL of freshly prepared 2 M NaOH solution, incubated for 30 min, and neutralized with 310 μL of concentrated HCl. The reaction was initiated by the addition of 1.5 mL of a solution of 2 mM potassium ferricyanide in 0.4 M phosphate buffer (pH 7.0) to 1.5 mL of the extract samples. Absorbance at 420 nm was recorded. Sinigrin was used as the standard (1, 10, and 50 μg/mL), and the results were expressed as µg of sinigrin equivalent (SE) per g of dry weight (DW):

C = (△OD − intercept)/slope × Vd/m

The TGC in the sample was calculated from the absorbance reading using a standard calibration curve of sinigrin. C is the concentration of glucosinolates in the sample (mmol/kg), OD is the change in optical density (420 nm), the slope and the intercept correspond to the calibration model for sinigrin, V is the volume (L) of ethanol used for extraction of glucosinolates, d is the dilution factor of the extract, and m is the mass of the sample.

### 3.6. Total Polyphenol Content (TPC)

The TPC of the cauliflower extract was determined by a previously reported method [24]. The results are reported as µg of gallic acid equivalents per g of dry weight (µg GAE/g DW), using gallic acid as the standard (1, 5, 10, 50, and 100 μg/mL). Briefly, 20 µL of the extract sample and 100 µL of Folin–Ciocalteu reagent were placed in tubes. The mixtures were incubated for 5 min before adding 80 µL of sodium bicarbonate. The absorbance of the mixtures was measured using a multiplate reader at 760 nm (Thermo Scientific, Waltham, MA, USA) after incubation for 120 min at room temperature.

### 3.7. Total Carotenoid Content (TCC)

A previously described method [34] was used to determine the TCC of the cauliflower extracts. Briefly, 1 mL of cauliflower extract was mixed with 1 mL of a hexane-diether solution (9:1 *v*/*v*), and the mixture was vortexed for 1 min. Then, 2 mL of saturated NaCl solution was added, and the mixture was vortexed for an additional 1 min. The mixture containing tubes were centrifuged at 3000× *g* for 5 min. The aqueous and organic layers were separated, and 0.5 mL of the organic layer was transferred to a new tube using a micropipette, and the solvent was removed using a nitrogen evaporation system. The dried extract was then redissolved in 2 mL of methanol, and 200 µL of the fraction was transferred to a 96-well plate to be measured spectrophotometrically at 450 nm. The results were expressed as µg β-carotene equivalence (BE)/g DW.

TCC = (A × V × 10^4^ × 4)/(A1% × W)

A = Absorbance at 450 nm

V = Total volume of dissolved extract (mL)

10^4^ = Conversion factor

4 = Dilution factor

Al% = Extinction coefficient for β-carotene

W = Weight of sample (g)

### 3.8. Ferric Reducing Antioxidant Power (FRAP) Assay

The total antioxidant capacity of the extracts was determined using a FRAP assay [30]. Briefly, to prepare the FRAP reagent, ferrous tripyridyl-triazine complex (Fe (III)-TPTZ) solution and hydrochloric acid (HCl) were added to 50 mL of distilled water. A 96-well plate was used to react 10 µL extract with 300 µL of FRAP reagent, and after 5 min incubation at 35 °C in the dark, the absorbance at 593 nm was measured using a TECAN plate reader based on Trolox equivalents (TE) (Infinite 200 PRO, TECAN, Mannedorf, Switzerland). The calibration curve was prepared using 5 to 450 µM Trolox.

### 3.9. DPPH Radical Scavenging Assay

The total antioxidant activity of the cauliflower extract was also measured using a DPPH radical scavenging activity assay. The assay of the extract was conducted according to a reported method [35]. Briefly, a 0.2 mM DPPH reagent was prepared, and 150 μL of DPPH reagent was added to 96-well plates. The extracts were dissolved in water to a concentration gradient (6, 3, 1.5, 0.75, 0.37 mg/mL), and 150 μL of each was added.

The following equation was used to calculate the inhibition percentage:

Antioxidant activity (% inhibition) = (Ab blank − Ab sample)/(Ab blank) × 100

where Ab sample is the absorbance value of the cauliflower extract, and Ab blank is the absorbance value without cauliflower extract. The antioxidant activity of the extracts was represented by the IC50, which is the concentration of the tested material needed to reduce the initial DPPH concentration by 50%. The IC50 value was determined using the % inhibition vs. antioxidant concentration curve in Microsoft Office Excel.

### 3.10. UPLC-ESI-MS Analysis of the Extracts

Quantification of major bioactive compounds present in upcycled kale was performed using the UPLC-ESI-MS, which consists of a Waters TQD-2 MS system (Micromass, Cary, NC, USA) and was managed with a MassLynx V4.2 data analysis software system. The samples used for UPLC analysis were filtered through 0.2 μm nylon filters and transferred into amber vials. Samples were injected into a UPLC system, equipped with a reverse-phased Waters HSS T3 column (2.1 × 100 mm, 1.7 μm) (Waters, Milford, MA, USA). For the determination of sulforaphane, a linear gradient profile with a flow rate of 0.4 mL/min of 0.1% formic acid in water (solvent A) and 0.1% formic acid in acetonitrile (solvent B) was used, with the proportion of solvent B changing over time (min); (time, solvent B%): (0, 8%), (1, 8%), (10, 99%), (11, 17.5%), and (12, 8%). The electrospray ionization source in positive mode (ESI+) with *m/z* 117.94 (retention time 4.51 min) was used with nebulizing gas (N_2_) at 200 °C (650 L/h), capillary voltage of 3500 V, and cone voltage of 30 V. For the determination of glucoraphanin, a linear gradient profile with a flow rate of 0.2 mL/min of 0.1% formic acid in water (solvent A) and 0.1% formic acid in acetonitrile (solvent B) was used, with the proportion of solvent B changing over time (min); (time, solvent B%): (0, 0%), (3, 0%), (6, 5%), (10, 90%), (11, 90%), and (12, 0%). The electrospray ionization source in negative mode (ESI-) with *m/z* 434.02 (retention time 1.35 min) was used with nebulizing gas (N_2_) at 200 °C (650 L/h), capillary voltage of 3500 V, and cone voltage of 52 V. The quantification was done with calibration curves created using external analytical standards (Figure S1).

### 3.11. Statistical Analysis

The Central Composite Design (CCD) with three factors (ethanol content, extraction temperature, and time) was generated and analyzed using Minitab Version 22 to determine the optimum setting of the factors that maximized the studied three response variables (TGC, TPC, and TCC from upcycled cauliflower extracts). The analyses included verifying the validity of model assumptions (normal distribution and constant variance assumptions on the error terms). The independence assumption on the error terms was validated by the random order of the 20 runs. This was followed by testing the significance of each model term, producing contour plots for each response variable, using a response optimizer to determine the optimum settings of the factors, and producing overlaid contour plots to determine the “sweet spot” that maximizes all three response variables. Prism 6 software (GraphPad Software, La Jolla, CA, USA) was used to draw the graph of comparison studies.

## 4. Conclusions

In this study, UAE was successfully optimized using RSM to enhance the extraction of glucosinolates using food-grade ethanol from upcycled cauliflower extract. The results of the CCD revealed that the optimal extraction conditions for maximizing TGC were found to be 42% ethanol, 43 °C extraction temperature, and 30 min extraction time. It was found that a lower ethanol concentration (≤50%), extraction temperature (≤50 °C), and shorter extraction times (≤40 min) resulted in higher glucosinolate content, while excessive increases in any of these parameters led to reduced yields. Based on the UPLC-ESI-MS analysis, the optimized UAE extracts contained glucoraphanin (1.31 μg/g DW of biomass) and sulforaphane (28.6 μg/g DW of biomass). The optimized extracts also possessed greater antioxidant activity as determined by FRAP and DPPH radical scavenging assays. This study suggests the UAE–ethanol as an efficient, environmentally friendly, and consumer-acceptable process for extracting valuable phytochemicals from upcycled cauliflower. Future studies should focus on evaluating the scalability and the therapeutic and preventive potential of upcycled cauliflower extracts for practical application in nutrition and health.

## Figures and Tables

**Table 1:** Central composite design (CCD) arrangement for ultrasonic bath extraction of phytochemicals from cauliflower.

Standard Run Order	Ethanol (%)	Time (min)	Temperature (°C)	TPC (µg GAE/g DW)	TCC (µg CE/g DW)	TGC (µg SE/g DW)
1	30	28	40	88.68	1.49	6633
2	70	28	40	99.36	2.57	3810
3	30	52	40	94.48	2.64	4395
4	70	52	40	103.7	2.81	104.8
5	30	28	60	87.72	2.16	4625
6	70	28	60	98.00	2.90	4216
7	30	52	60	85.96	2.08	4542
8	70	52	60	85.55	2.95	639.0
9	16.4	40	50	80.54	2.50	4780
10	83.6	40	50	98.64	1.52	5130
11	50	20	50	98.12	1.42	5733
12	50	60	50	87.57	2.03	5894
13	50	40	33.1	85.50	1.73	6767
14	50	40	66.8	104.3	2.46	4234
15	50	40	50	106.5	2.59	5810
16	50	40	50	103.8	3.51	5617
17	50	40	50	116.7	4.17	5887
18	50	40	50	105.4	4.08	6190
19	50	40	50	99.64	4.22	6193
20	50	40	50	100.1	4.11	5685

Note: the CCD natural values of ethanol concentration, extraction time, and temperature are shown. TPC: total polyphenol content; TCC: total carotenoid content; TGC: total glucosinolate content; GAE: gallic acid equivalents; SE: sinigrin equivalents; CE: beta-carotene equivalents.

**Table 2:** Analysis of variance (ANOVA) *p*-values that show the significance of the coefficients of the regression model used for the CCD design on TGC, TPC, and TCC.

Source of Variation	TPC (µg GAE/g DW)	TCC (µg CE/g DW)	TGC (µg SE/g DW)
Temp	0.927	0.500	0.398
Time	0.433	0.377	0.142
% ethanol	0.047 **	0.647	0.094 *
Temp × Temp	0.101	0.030 **	0.246
Time × Time	0.054 *	0.009 ***	0.357
% ethanol × % ethanol	0.019 **	0.021 **	0.126
Temp × Time	0.259	0.491	0.622
Temp × % ethanol	0.631	0.856	0.546
Time × % ethanol	0.566	0.700	0.293
R^2^	67.93%	69.34%	57.52%

TGC: total glucosinolate content; TCC: total carotenoid content; TPC: total phenolic content; * indicates marginally significant (at 10% level of significance); ** indicates significant (at 5% level of significance); *** indicates highly significant (at 1% level of significance). Temp: temperature; % ethanol: ethanol concentration.

**Table 3:** The glucosinolate content of cauliflower extract using UPLC-ESI-MS.

Extraction Conditions	Glucoraphanin (μg/g DW)	Sulforaphane (μg/g DW)
42% ethanol–UAE with RSM optimized conditions	1.31 ± 0.12 ^a^	28.6 ± 3.34 ^a^
42% methanol–UAE with RSM optimized conditions	1.21 ± 0.04 ^a^	24.3 ± 0.92 ^a^
70% acetone–maceration at 70 °C for 20 min	1.08 ± 0.17 ^ab^	5.47 ± 0.22 ^b^
70% methanol–maceration at 70 °C for 20 min	0.88 ± 0.13 ^b^	0.88 ± 0.08 ^c^

Note: Letters indicate significant differences (*p* < 0.05) based on the Tukey method. DW, dry weight of biomass.

## Data Availability

Data are contained within the article.

## Supplementary Materials

The following supporting information can be downloaded at: https://www.mdpi.com/article/10.3390/molecules30112326/s1, Figure S1: Liquid chromatography chromatograms of sulforaphane (*m/z* of 177.94 and retention time of 4.51 min) and glucoraphanin (*m/z* of 434.02 and retention time of 1.35 min) in single ion mode.
