# Supplementary material for: Optimization of Ultrasound-Assisted Extraction of Glucosinolates from Upcycled Cauliflower Using Response Surface Methodology
Source: Molecules. 2025 May 26;30(11):2326. doi: 10.3390/molecules30112326 (PMC12156931; doi:10.3390/molecules30112326)
Supplement: Supplementary file 1 [file molecules-30-02326-s001.zip › molecules-3649856-supplementary.pdf]

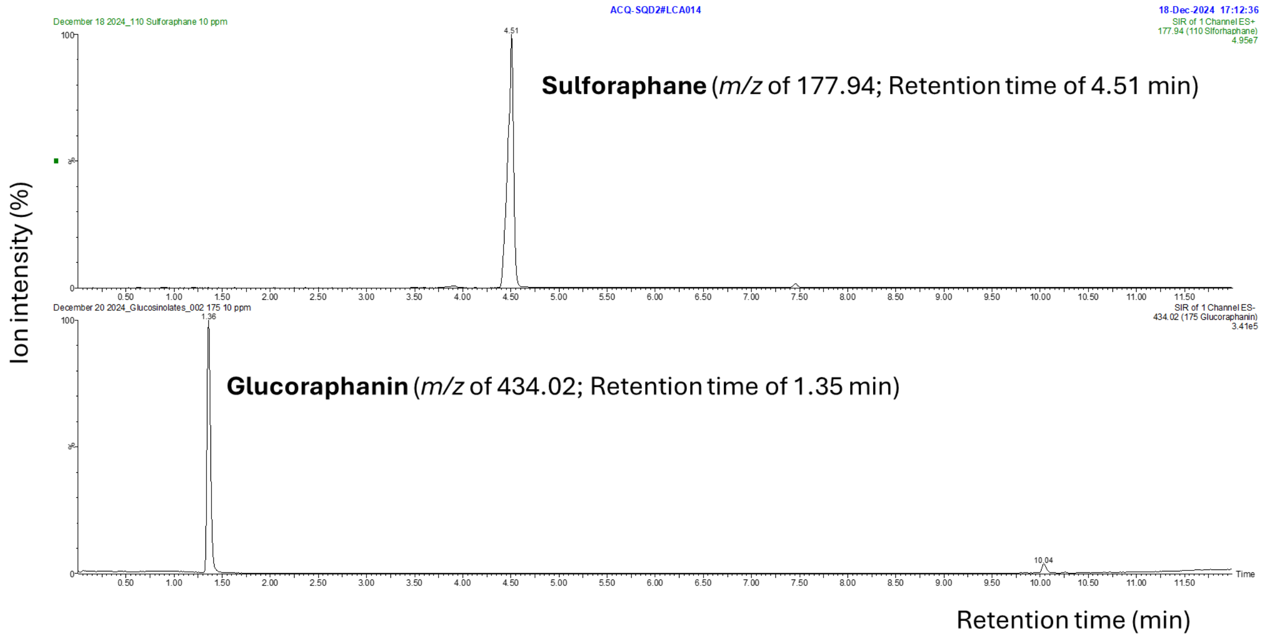

Figure S1. Liquid chromatography chromatograms of sulforaphane (*m/z* of 177.94 and retention time of 4.51 min) and glucoraphanin (*m/z* of 434.02 and retention time of 1.35 min) in single ion mode.
